# A Final Frontier in Environment-Genome Interactions? Integrated, Multi-Omic Approaches to Predictions of Non-Communicable Disease Risk

**DOI:** 10.3389/fgene.2022.831866

**Published:** 2022-02-08

**Authors:** Alexandra J. Noble, Rachel V. Purcell, Alex T. Adams, Ying K. Lam, Paulina M. Ring, Jessica R. Anderson, Amy J. Osborne

**Affiliations:** ^1^ Translational Gastroenterology Unit, Nuffield Department of Experimental Medicine, University of Oxford, Oxford, United Kingdom; ^2^ Department of Surgery, University of Otago Christchurch, Christchurch, New Zealand; ^3^ School of Biological Sciences, University of Canterbury, Christchurch, New Zealand

**Keywords:** multi-omics, integrated analyses, non-communicable diseases (NCD), gene-environment inreractions, health outcomes, disease risk prediction

## Abstract

Epidemiological and associative research from humans and animals identifies correlations between the environment and health impacts. The environment—health inter-relationship is effected through an individual’s underlying genetic variation and mediated by mechanisms that include the changes to gene regulation that are associated with the diversity of phenotypes we exhibit. However, the causal relationships have yet to be established, in part because the associations are reduced to individual interactions and the combinatorial effects are rarely studied. This problem is exacerbated by the fact that our genomes are highly dynamic; they integrate information across multiple levels (from linear sequence, to structural organisation, to temporal variation) each of which is open to and responds to environmental influence. To unravel the complexities of the genomic basis of human disease, and in particular non-communicable diseases that are also influenced by the environment (e.g., obesity, type II diabetes, cancer, multiple sclerosis, some neurodegenerative diseases, inflammatory bowel disease, rheumatoid arthritis) it is imperative that we fully integrate multiple layers of genomic data. Here we review current progress in integrated genomic data analysis, and discuss cases where data integration would lead to significant advances in our ability to predict how the environment may impact on our health. We also outline limitations which should form the basis of future research questions. In so doing, this review will lay the foundations for future research into the impact of the environment on our health.

## Introduction

Genomics is revolutionising our understanding of the biological basis of disease, and it is undisputed that our individual genotypes, in combination with lifetime exposures (our environments), are critical determinants of non-communicable disease (NCD) risk. For example, it is well established that the prenatal and early life environments strongly influence the risk of non-communicable disease in later life [the Developmental Origins of Health and Disease, DOHaD (Barouki et al., 2012)], This is exemplified in a recent study into the role of genetics, early life nutrition and their interaction on adult health, which demonstrated that high genetic risk for non-communicable disease can be mitigated by an environmental intervention [i.e., longer duration of breastfeeding (Wang et al., 2021)]. However, definitively connecting environmental exposures to specific health outcomes remains stubbornly challenging, and it is generally unclear whether the associations we see are causal, partly causal, or simply correlative. While techniques such as causal inference and Mendelian randomisation have improved our ability to determine putative causal relationships between risk factors and disease [e.g., (Timpson et al., 2005; Pingault et al., 2018)], there is currently no way to predict whether, or how, a particular environmental exposure may help or harm human health, in a way that gives us the ability to assign cause and effect. Thus, mitigating the impacts of the environment on our health largely remains an elusive goal.

One factor that contributes to our lack of power to detect cause and effect for environment-phenotype interactions, is that the impact of our environment on our genome is governed by much more than our genotypes (Manolio et al., 2009). For example, there are complex layers of information embedded in the structure of the genome and epigenome that impact gene expression. Due to the complex nature of the interactions between these information levels and the other nuclear and cellular processes that emerge within the complex system, we still do not fully understand what happens at the genomic level to translate environmental signals into phenotypes, and very rarely do we have the power to draw conclusions, because, while the genetic code is largely static, the multiple, dynamic, and interacting layers of genome structure and organisation are open to environmental influence. Thus, while it is clear that there is a distinct sequence of events that must happen in order to translate environment into phenotype, exploration of these events, and any attempt to predict the effects of the environment on our health, will require integration of information from across multiple biological and information levels. For example, quantifying DNA methylation in response to a particular environmental variable will only provide an indication that something biologically meaningful might be happening at a few loci in the genome—it may well mark biologically relevant pathways, but it cannot tell us the impact that methylation has on gene expression. Therefore, we struggle to predict the phenotypic impact of DNA methylation alone. Many complex biological questions, such as understanding the biological basis of environment-driven health inequalities, have not been addressable with one-dimensional reductionist approaches, and advances in our ability to predict the impact of our environment on our health therefore will require integration of multi-layered genomic data in a way that accounts for interactions between and across the biological layers.

Developments in ‘omics techniques and technologies, and environmental electronic data (e.g., from wearables) means that we are at a point in our endeavours where we can explore an integrated, multi-omics approach to health and wellbeing. Here, we first describe the major and most well studied layers of genome regulation, and focus on the application of these to NCDs and complex disease, reviewing current efforts to integrate multi-omic data in disease. We describe new and emerging technologies that will improve our ability to assign a phenotypic impact to an environmental exposure. In doing so, we argue that progress in this field will be dependent on our ability to undertake integrated, multi-omic approaches that fully explore the environmental and molecular basis of complex disease.

### How Our Genomes are Regulated—Potential Areas for Environmental Influence

Perhaps one of the most well-defined epigenetic signals for gene/genome regulation is DNA methylation. DNA methylation is a common form of epigenetic genome regulation, wherein methyl groups are added to the 5’ positions of cytosines in cytosine-guanine dinucleotides (CpGs), which further correlates with histone modifications and chromatin accessibility. Importantly, patterns of DNA methylation can be altered by environmental exposures (Jaenisch and Bird, 2003) and we know that they can be influenced by early-life environment (*in utero* and early post-natal), which, itself, is associated with variation in later-life disease susceptibility (Gluckman et al., 2008; Barouki et al., 2012; Lillycrop and Burdge, 2012). However, while changes in DNA methylation are often identified in response to a changing environment (Feil and Fraga, 2012), methylation by itself does not explain the full complexity and diversity of the genomic response to the environment (Freeman et al., 2016). That is because DNA methylation is just one type of epigenetic signal that can work to regulate gene expression (Jaenisch and Bird, 2003; Jirtle and Skinner, 2007; Bonev and Cavalli, 2016). Other mechanisms include non-coding RNA (ncRNA) transcription (Jaenisch and Bird, 2003; Weber et al., 2007) and modification of histone protein tails within nucleosomes, both of which directly affect 3-dimensional (3D) genome organisation and, ultimately, nuclear functions (Risca and Greenleaf, 2015; Bonev and Cavalli, 2016).

The 3D organisation of the genome emerges from the sum of the functions that are occurring within the nucleus, and is widely considered to have a role in the regulation of gene expression (Cremer and Cremer, 2001; Lieberman-Aiden et al., 2009). For example, DNA looping brings distant gene enhancers and promoters together, which promotes the recruitment of RNA polymerase and ultimately gene transcription. Chromosomes are organised into highly conserved territories (Dixon et al., 2012; Sexton et al., 2012) and at a finer scale, precise domains, termed topologically associating domains (TAD). Genes located in the same domain are often co-expressed and are insulated from genes in neighbouring domains by domain boundaries (Nora et al., 2012). Perturbations of domain boundaries can disrupt both short- and long-range genomic interactions, sometimes with pathological outcomes (Franke et al., 2016). 3D genome organisation and chromatin accessibility can be studied using techniques such as ATAC-seq [Assay for Transposase-Accessible Chromatin with high-throughput sequencing (Buenrostro et al., 2015)] and proximity ligation experiments such as Hi-C (Bickmore and van Steensel, 2013; Stevens et al., 2017). Understanding 3D genome structure is important because chromatin remodelling is a dynamic and often adaptive response to the environment (De Nadal et al., 2011; Matilainen et al., 2017). For example, exposure to inhaled industrial chemicals (Fang et al., 2014) or heat stress (Petesch and Lis, 2008) results in alterations to chromatin structure, changing chromatin accessibility, with associated downstream effects on gene expression. 3D genome organisation has recently been implicated in the pathogenesis of obesity and diabetes (Fadason et al., 2017), highlighting the importance of integrating spatial information into interrogations of the genetic basis of complex disease.

Non-coding RNAs (ncRNAs) are transcribed from DNA but not translated into protein. Despite this, ncRNAs have broad roles in genomic regulation. For example, microRNAs (miRNAs) guide argonaute proteins to degrade mRNAs containing sequence targeted by the seed region of the miRNA, culminating in transcriptional silencing (Peters and Meister, 2007). ncRNA transcription can be altered by environmental factors (Saxena and Carninci, 2011; Tani et al., 2014) to directly influence gene expression patterns (Guttman and Rinn, 2012; Engreitz et al., 2016). Further, DNA methylation can influence ncRNA transcription to produce health-related phenotypic effects (Lujambio et al., 2010), suggesting the two processes can work together. Importantly, ncRNA interacts with chromatin and can alter the accessibility of genomic regions for transcription (Castel and Martienssen, 2013), and can remodel 3D genome structure (Cubeñas-Potts and Corces, 2015; Dekker and Misteli, 2015; Engreitz et al., 2016; Rowley and Corces, 2016).

Histone protein tails can be modified by post-translational modifications that include the addition of either acetyl groups or methyl groups (Bannister and Kouzarides, 2011), which can affect chromatin accessibility and alter transcription profiles. Patterns of histone modification can be explored by techniques such as mass spectrometry (MS) and chromatin immunoprecipitation and sequencing (ChIP-seq) (Esteller, 2007). Histone modifications can interact with DNA methylation, and this interaction has been associated with disease phenotypes [e.g., cancer (Vaissière et al., 2008)], as both are sensitive to the environment (Jirtle and Skinner, 2007; Dai and Wang, 2014). Therefore, histone modifications, and chromatin accessibly, are strong determinants of gene expression profiles.

In addition to the more traditionally recognised records of environmental impact on the genome, there are other sources of information that reflect and respond to the interactions between the host genome and environment. For example, the microbiome, proteome and metabolome each emerge from the complex web of environment, genetic, structural and epigenomic interactions. It is clear that perturbations of these systems can indicate an effect on health, which can be interrogated under an ‘omics platform, and integrated into subsequent analyses:

The human microbiome is generally defined as the ‘complete set of genes and genomes of the microbiota (bacteria, archaea, eukaryotes, and viruses) that reside in and on a person’. More extensive definitions also include aspects of the surrounding environmental conditions in their definition (Marchesi and Ravel, 2015). Microbiomes can be analysed at different levels, be that their metagenome (DNA) to assess community composition and functional capacity, or at the metatranscriptome (RNA) level; at this level, RNA is used to define community composition as well as characterise the activity of the organisms at the time of sampling. The microbiome, and by extension, the metagenome and metatranscriptome, is variable, depending on many environmental factors, such as anti- and probiotic use, age, diet, environment and physical activity levels. Despite few causal examples, it is widely recognized that changes in the gut microbiota are associated with the onset and progression of non-communicable diseases [reviewed in (Noce et al., 2019)], including autoimmune diseases such as multiple sclerosis (MS) and rheumatoid arthritis [RA, (Tsai et al., 2021)]. Comprehensive investigations of the microbiome are, by their nature, integrative requiring analyses of the metagenome and metatranscriptome; direct and indirect interactions between the microbiome and the host, and environment-microbe-metabolism interactions (Kurilshikov et al., 2019).

The proteome is the complete set of proteins expressed by an organism, tissue or cell at a particular point in time. Naturally, the proteome shares components of its dynamism with the transcriptome and the epigenome as a result of the process of gene expression. However, the proteome is widely recognized as not having a 1:1 relationship with the transcriptome, due to factors affecting translation and post-translational modifications [e.g., (Ghazalpour et al., 2011; Wang et al., 2019)]. Therefore, a complete picture of cellular activity cannot be determined from the transcriptome alone. Recent studies connect the proteome to immune dysregulation and obesity (Garrison et al., 2017) and traits relevant to the DOHaD hypothesis (Sarli et al., 2021). Moreover, the proteome is known to respond to environmental stimuli (Koga et al., 2011; Calamini and Morimoto, 2012) including diet (Vileigas et al., 2019), chemical exposure (Lee et al., 2018) and smoking (Sinha et al., 2021). Therefore, given the often imperfect correlation between the transcriptome and the proteome, proteomic data is one layer of omic information that adds value to integrated, multi-omic approaches; it allows for the refinement of the number of target genes deemed necessary to investigate further, since gene expression changes that are not correlated with a coordinated change in protein expression can be discounted from downstream analyses.

The metabolome comprises all current low molecular weight cellular metabolites, indicating current cellular activity levels. The metabolome essentially denotes the end product of cellular processes, allowing a functional readout of an organism (Wang et al., 2020). Altered metabolomes have been identified in many NCDs, including Type II diabetes and obesity (Fiehn et al., 2010; Zhang et al., 2014; Merino et al., 2018). Furthermore, hormones and other metabolites can be programmed *in utero* through epigenetic mechanisms (Rauschert et al., 2017), such that a child’s metabolism can be influenced by its environmental exposures (Cottrell and Ozanne, 2008). This means that the metabolome may contribute to our understanding of the developmental basis of disease by refining our ability to assign functional changes to environmental exposures. Recent studies also demonstrate the value of integrating metabolome with microbiome data to profile disease pathogenesis, for example, a recent review of autoimmune disease describes how the microbiome and associated metabolic profile are altered by ‘modern’ lifestyles, which is impacting on inflammatory responses (Tsai et al., 2021). Given that the metabolome itself is the end product of cellular processes, it stands to reason that, like the proteome, it can be indirectly altered by environmentally-induced genomic, epigenetic, structural and microbiotic changes. This means that integration of the microbiome in multi-omics studies will provide indications of the functional significance of observed genomic and epigenetic changes, which may highlight mechanistic pathways that are important for the aetiology of disease.

Lastly, it is important to also consider the implication of an individual’s underlying genetic variation, and its interaction with environmental factors, when assessing the impact of the environment on genome regulation. For instance, we know that GWAS explain only a portion of the heritability of NCDs such as obesity and MS (Silventoinen et al., 2016; International Multiple Sclerosis Genetics Consortium, 2019) and that GWAS cannot explain all the variability of traits; many causative loci exist in intergenic regions of the genome (Manolio et al., 2009), and further, disease heritability has been observed to interact with an individual’s environment [e.g., (Hüls et al., 2021; Jacobs et al., 2021; Ye et al., 2021)]. Therefore, since underlying genetic variation may influence, e.g., methylation patterns if that variation is at a modifiable cytosine residue, genetic variation cannot be discounted when attempting to predict the phenotypic effects of our environments.

How do the layers of complexity interact to influence phenotypes? Reductionist approaches that do not integrate these different levels of information may miss many of the crucial interactions that determine how our genomes orchestrate a biological response to our environments. In so doing, we will lessen our ability to investigate the effects of our environmental exposures and lose our power to predict how they might be influencing our health.

### How Research is Exploring Integrated Approaches to Understanding the Impact of the Environment on Disease

Non-communicable diseases are diseases that are non-infectious in nature, but nevertheless cause severe and debilitating disease, and are a major public health burden and cause of morbidity and mortality (W.H. Organization, 2019). Perhaps due to our ‘transition to modernity’ (Corbett et al., 2018) such diseases are increasing in prevalence globally, making them the focus of intense research. Here, we focus on examples of the application of integrated, multi-omic approaches to several NCDs that are all themselves a product of the interaction between environmental exposures and genetic predisposition.

### Obesity

Obesity is by far the most prevalent NCD for which data on integrated, multi-omic approaches exist. This is because obesity is a major public health burden, increasing in prevalence (Abarca-Gómez et al., 2017) and is a risk factor for many other metabolic diseases such as type II diabetes, cardiovascular disease, and some cancers (Johnson et al., 2015; Weihrauch-Blüher et al., 2019). Obesity is driven by a combination of an underlying genetic predisposition, and environmental factors (Albuquerque et al., 2017), including *in utero* exposures (Tounian, 2011). This means that unpacking underlying genetics, maternal and individual environmental effects to determine which environmental impacts are causative, versus those that are correlational, is difficult.

A handful of studies demonstrate an integrated approach, not necessarily on the impact of the environment, but rather, exploring multiple layers of genomic data to detect genomic changes relevant to a phenotype. For example, a recent study by van der Kolk et al. (van der Kolk et al., 2021) investigated the link between obesity and metabolic complications through the application of RNA sequencing, proteomics and metabolomics; their study cohort contained 49 BMI-discordant monozygotic twin pairs, meaning their shared genetic background enabled the researchers to build a metabolic and genomic profile of acquired (environmentally-dependent) obesity. The authors detected a downregulation of mitochondrial pathways and an upregulation of inflammatory pathways, along with alterations to the metabolome that were specific to acquired obesity. However, while this is a strong example of investigations of multiple types of genomic data, these data are not strictly integrated in their analyses. Rather, Kolk et al. present these data side-by-side as independent profiles in a manner that reinforces the biological interpretations without achieving true integration as a means of tracing cause and effect.

Integration, has been attempted in other obesity studies. For example, Chen et al. (Chen et al., 2021), citing a recent epigenome-wide association study that linked individual CpG sites with obesity traits (Sayols-Baixeras et al., 2017), explored the correlation between DNA methylation and gene expression. Their study reported associations between genes that were differentially expressed and differentially methylated. They also identified two novel genes, S100A8 and S100A9, expression of which correlated negatively with methylation and were associated with increased obesity. This study highlights the strength of integrating DNA methylation and gene expression data to deepen our understanding of the relationships between DNA methylation and gene expression in complex phenotypes.

Many genomic analyses are applied to human studies retrospectively as part of post-hoc analyses, and many are also limited in their scope, in terms of type of data available, tissue of origin, and cohort size. Unsurprisingly, then, we can gain more insight into integrated multi-omic approaches using models of human disease. For example, Joslin et al. (Joslin et al., 2021) recently attempted to functionally interpret genome-wide association study data in obesity, by capturing information on chromatin accessibility, gene expression, and long-range enhancer-promoter interactions, in human-induced pluripotent stem cell (iPSC)-derived hypothalamic neurons. Their data indicated that the genetic architecture at GWAS loci is complex, but nevertheless they were able to detect putative enhancer-modulating variants that have regulatory properties in their cell line, at obesity-related loci. The strength of their highly integrated approach, and the increase in scope offered by using a cell line (therefore only a ‘single’ genome) has allowed them to develop a pipeline to prioritize GWAS target genes for functional follow up, potentially limiting the number of functional loci that further studies may need to investigate in human-based cohorts. Joslin et al.’s study highlights the potential of integrating multiple layers of genomic complexity. The expansion of their pipeline to include, e.g., DNA methylation data, along with environmental variables that drive epigenetic variation between individuals, could drive further discoveries in this area, by facilitating a clearer understanding of how epigenetic mechanisms contribute to the association between SNPs and enhancer-promoter interactions.

These examples (Chen et al., 2021; Joslin et al., 2021) demonstrate a role for integrated genomic analyses in the relationship between the environment and a phenotype. As more data is generated, and as techniques improve, finding a way to integrate environmental variables into models of integrated multi-omic approaches to obesity will be a key driver of our ability to assign causation to both environmental factors and genetic and epigenetic mechanisms in the development of obesity. This is because an individual’s specific environment can tell us something meaningful about the exposures that may be driving differences at the cellular level, that may be impacting on, e.g., gene expression, the microbiome, and the proteome. Current computational capabilities and research methodologies suggest that the further integration of 3D genome structure, to aid in the linking of risk variants to target genes (Krijger and De Laat, 2016), and the unequivocal role of the microbiome in obesity, (Hartstra et al., 2015; Jayasinghe et al., 2016; Maruvada et al., 2017), is a natural focal point for future research in this disease.

### Type II Diabetes

Type II diabetes (T2D) is characterised by a resistance to insulin, meaning that blood glucose levels in the body are not able to be controlled properly, often leading to hyperglycemia, obesity, hypertension and hyperlipidemia, and eventually severe complications such as blindness and kidney failure (W.H. Organization, 2016; Khawandanah, 2019). T2D is becoming a global epidemic (Kaiser et al., 2018), thus, understanding environmental drivers of T2D, and how they may interact with an individual’s underlying genetics to cause disease, will be fundamental to a global approach to mitigate its rise in prevalence.

In a meta-analysis of diabetes GWAS, Schierding et al. (Schierding and O’Sullivan, 2015) integrated SNP data, 3D genome and eQTL data, to identify ‘spatial hubs’, or connections between loci in genes that contribute to disease. Additionally, Xue et al. (Xue et al., 2018) combined data from gene expression studies of human blood with GWAS and identified a suite of putative functional genes for T2D, linking GWAS data with a potential functional (gene expression) output. Further, Xue’s research integrated of DNA methylation data with epigenome annotation data and identified three genes (*CAMK1D, TP53INP1, ATP5G1*) as having a plausible regulatory mechanism in T2D. In the context of this review, these findings are instrumental for their ability to refine large (e.g., GWAS) datasets and improve their predictive power, by associating disease-associated SNPs with downstream and integrated layers of genomic regulation (Schierding and O’Sullivan, 2015), and by the integration of SNPs with gene expression and epigenome annotation data (Xue et al., 2018). These approaches could be readily applied to other NCDs, with environmental covariables included where studies allow, for example, integrating lifestyle and family data; accounting for heritable genetic variation and lifestyle risk factors will to strengthen the ability to assign causation to a particular risk factor (environment/lifestyle or genetic).

There are multiple examples of GWAS to determine susceptibility loci for T2D. However, the large majority of the loci identified fall in non-coding regions of the genome, meaning that it can be highly challenging to determine which genes and transcripts their variation is relevant to, and which molecular pathway they may influence. Integrated multi-omic approaches are valuable to attempt to predict which disease-associated loci are functionally relevant, in the context of the phenotype of interest. This is important when considering environmental drivers of complex disease, because if individual variation at a particular locus is associated with an environmentally-influenced disease, determination of the functional impact of that locus may help us predict whether that locus may be causative for disease, or simply correlated. For instance, that locus may mark an underlying CpG site, or be located within a ncRNA sequence, which we know are sensitive to environmental influence, and therefore may influence the expression of genes that may be phenotypically relevant. To this end, efforts have been made to develop analytical pipelines that integrate genetic, genomic and biological data to produce networks that indicate connectivity between GWAS loci and candidate causal genes. For example, Nyaga et al. (Nyaga et al., 2021) used integrated genomics to ask whether there were any shared genetic features of type I diabetes (T1D) and T2D; their functional approach integrated Hi-C and eQTL data to characterise the functional impacts of disease-associated SNPs, identifying genetic regulatory regions that alter regulation of genes common to both T1D and T2D, that are associated with disease development. Additionally, Fernández-Tajes et al. (Fernández-Tajes et al., 2019) present an analytical pipeline to define the transcriptional activity of T2D-associated SNPs, integrating genomic data to reveal connectivity between candidate genes at T2D GWAS loci. These approaches, while distinct, can be applied to other diseases, using other types of genomic data, thereby providing insights into the diseases that are identifying new means of stratification, prevention and treatment, which collectively prove the importance of these types of approaches.

Mens and colleagues (Mens et al., 2020) used large-scale GWAS data to detect variants associated with T2D traits, and integration of DNA methylation and miRNA expression data confirmed that several of these miRNAs were associated with T2D traits. The data used by Mens et al. was obtained from human peripheral blood, therefore their identified miRNAs could be considered as biomarkers for T2D. Their study highlights yet another strength of integrated analyses; the computational reduction of a huge study into practical targets by assigning a more likely function to those targets, prioritising areas for follow up.

### Multiple Sclerosis

Multiple sclerosis (MS) is an autoimmune disorder that affects the central nervous system (CNS). It leads to the destruction of myelin in the CNS, blocking the transport of signals along the nerves to the brain, meaning that movement, sensation and body functions are impacted. MS is characterised by periods of recovery and relapse over a number of years, before ending in disability, with no treatment available at the progressive disease stage.

The International Multiple Sclerosis Genetics Consortium, and others, have published numerous GWAS to identify genetic factors that contribute to MS [e.g., (Consortium et al., 2007; International Multiple Sclerosis Genetics Consortium, 2019; Mo et al., 2019)], however, there are also multiple known environmental risk factors for MS development, most prominently, lack of vitamin D exposure, smoking, and exposure to Epstein-Barr virus (EBV) (O’Gorman et al., 2012). For example, a recent longitudinal study in military veterans demonstrated a strong link between EBV and MS (Bjornevik et al., 2022). Despite these known associations, and the complex interplay between genetics and the environment, most studies of MS focus only on GWAS, or are conducted at the candidate-gene level, for example, correlations between promoter methylation and gene expression levels (Hosseini et al., 2020). A small number of studies have started to integrate genomic information across multiple genome technologies and layers of regulation. For example, Gokuladhas and colleagues (Gokuladhas et al., 2020) integrated SNPs from (amongst other neuromuscular disorders) MS patients with Hi-C and eQTL data, to identify target genes to prioritise for therapy and treatment of MS. Their approach, essentially determining SNP-mediated gene regulation, highlights the potential for the integration of SNP and spatial data for more precisely identifying the molecular mechanisms of complex disease, as well as providing evidence of disease-related SNP functionality, particularly given that most SNPs are in intergenic regions of the genome. Gokuladhas et al. have since expanded the approach to include protein-protein interaction data, and applied it more widely to autoimmune diseases to reveal shared biological processes across autoimmune diseases (Gokuladhas et al., 2021).

Mo et al. (Mo et al., 2019) employed Mendelian randomisation to explore GWAS, gene expression (eQTL) and epigenome-wide association study (mQTL) data, to determine whether e- and mQTL data, in combination with GWAS, was a viable way to prioritise relevant GWAS loci for further investigation. While not integrated in the strict sense (the authors explored overlap and validation in the individual datasets) this technique was highly successful in identifying potentially causal SNPs and DNA methylation differences, demonstrating the strength of this methodology to identify genomic features that may participate in the pathogenesis of MS.

Rather than interrogating genomic loci such as SNPs, Cervantes-Gracia and others (Cervantes-Gracia and Husi, 2018) used publicly available expression datasets to identify the most common molecules relevant to MS. Their approach was to generate interaction networks to identify molecular pathways/conserved networks that are deregulated across MS. They integrated mRNA and miRNA expression profile datasets, and impressively, combined these with differentially expressed genes identified through studies of, e.g., EBV infection, allergies and other autoimmune diseases. Their research uncovered a suite of molecules (mRNAs, miRNAs) that were correlated and deregulated in their datasets, that they could use to infer novel findings about the primary cause of the molecular changes seen in MS blood samples.

Based on evidence gathered from research into other NCDs, namely, that an integrated, multi-omic approach is valuable and insightful, together with the paucity of such approaches being applied to MS, highlights how much MS research will benefit from the integration of multiple layers of genomic data, particularly in light of the strong and well-identified environmental factors [e.g., (Bjornevik et al., 2022)]; this approach will allow us to interrogate the impact of the environment and the genome on MS progression, providing novel insights into the biological basis of disease development and progression.

### Alzheimer’s Disease

Alzheimer’s disease (AD) is the most common type of dementia, a form of brain degeneration that ablates memory and cognitive functions. AD is increasing in prevalence, most likely due to longer life expectancies globally, and it is estimated that 100 million people will have AD or dementia by the year 2050 (Palmqvist et al., 2020). There is currently no treatment to prevent the progression of dementia (Mehta et al., 2017), although some drugs can help to manage symptoms, if detected early. However, because AD is usually only diagnosed at an advanced age, early diagnosis and rapid treatment is challenging.

Alzheimer’s disease has been the focus of multiple GWAS over recent years, with over 20 independent loci associated with the disease (Van Cauwenberghe et al., 2016). Further, there are known environmental risk factors that associate with a diagnosis of AD, such as obesity, hypertension and tobacco smoking (Østergaard et al., 2015). Because AD is currently incurable, understanding the environmental and genetic determinants of AD is paramount if we wish to be able to both prolong life *via* early diagnosis, and develop effective and additional therapies, and integrated, multi-omic studies are the clear pathway to achieving this. Currently, genomics, transcriptomics, proteomics and metabolomics are offering a more comprehensive view of molecular pathways underlying the development of neurodegenerative diseases. For example, they are helping to differentiate subtypes of patients based on their specific molecular signatures, to aid individual treatment plans for patients (La Cognata et al., 2021). Additionally, genomic technologies are profiling the transcriptome of the brain with neurodegenerative diseases (Neff et al., 2021), and while studies explore the ‘omics’ of AD, few have done so in an integrated manner, to improve the power of their associations.

Thus, as with many complex diseases, truly integrated, multi-omic studies are scant. However, a recent comprehensive study by Nativio and colleagues (Nativio et al., 2020) used transcriptome profiling of human brain samples to inform proteomic analysis and ChIP-seq, followed by an exploration of the overlap of their identified genes, with GWAS and eQTL data. This powerful study identified upregulation of transcription- and chromatin-related genes (including the histone acetyltransferase genes for H3K27ac and H3K9ac) in AD, culminating in the new knowledge that the histone modifications H3K27ac and H3K9ac and genome reconfiguration are potentially important AD. Further, multi-omic atlases of AD from human brain tissue are currently being constructed (De Jager et al., 2018), that include genotypes, whole genome sequencing, DNA methylation, chromatin immunoprecipitation, RNA and miRNA profiles, with the focus of understanding the molecular mechanisms of AD in the target organ, rather than a cell line or animal model. Nativio’s study suggests that we can use integrated data to explore genomic mechanisms associated with AD, and genome atlases will allow us to integrate data across multiple levels. This is important in an uncurable disease such as AD, where the identification of targets and the development of new therapies is imperative.

### Rheumatoid Arthritis

Rheumatoid arthritis (RA) is a chronic inflammatory autoimmune disease that affects joints. It is characterised by increased inflammation in the synovial membrane, which causes swelling and damages the joint *via* bone erosion. Like other autoimmune diseases, RA is characterised by periods of flare/exacerbation and of remission, and, again like other autoimmune diseases, its aetiology is a complex mix of underlying genetic risk factors (e.g., particular HLA class II genotypes (Raychaudhuri et al., 2012) and environmental risk factors [age, *in utero* exposure to tobacco, personal tobacco use, obesity, and a high sodium diet (Deane et al., 2017)]. There is no cure for RA, but joint destruction can be delayed by prompt and aggressive anti-inflammatory treatment.

Thus, because of the known genetic determinants of RA that lead to increased susceptibility, which is further enhanced by the multiple environmental risk factors, that are themselves all able to modify the epigenome [e.g., (Joubert et al., 2012; Besingi and Johansson, 2014; Florath et al., 2014; Jaffe and Irizarry, 2014; Ivorra et al., 2015; Sayols-Baixeras et al., 2017; Noble et al., 2021)], researchers are applying multi-omics approaches to identify networks that drive disease progression, and to prioritise candidate genes for study, all of which may aid in the identification of targets for novel therapeutics. For example, Whitaker et al. (Whitaker et al., 2015) used an unbiased approach to integration (i.e., they did not assume a relationship between DNA methylation and gene expression) to prioritise candidate genes; they devised a strategy to identify ‘multi-evidence genes’ (MEGs) to identify triple-evidence genes that overlap between epigenome, transcriptome and sequence data, to collate sets of genes that were implicated in RA. Their approach identified seven triple-evidence genes, validating some as candidates for new RA therapies. Further, an assessment of RA pathogenesis was undertaken *via* an integrated DNA methylation and gene expression approach by Li Yim and colleagues (Li Yim et al., 2021); they identified a suite of 59 genes with coordinated changes at the gene transcript and DNA methylation level, which were associated with immune response pathways. Their research provided more evidence for molecular changes associated with RA pathogenesis, and their approach, like that of Whitaker et al., will be useful in aiding in the prioritisation of targets for new therapeutics, *via* the identification of potential new drug targets. Another benefit of a multi-omic approach is that it provides the power to interrogate disease-relevant tissue in a dynamic way, allowing a fuller understanding of the variants that shape disease. A relevant example of this is that of Ha et al. (Ha et al., 2021), who explore GWAS, gene expression and DNA methylation in CD4+ T cells in patients with RA; CD4+ T cells are the most disease-relevant tissue in RA. Their research identified a larger number (2575) of RA-specific differentially expressed genes that correlated with RA-specific differentially methylated regions of the genome, and that were enriched in T cell differentiation and activation pathways. They were also able to show, through their multidimensional approach, that many of the differentially expressed genes were explained by eQTLS (771, for transcripts) and mQTLs (83, for differentially methylated regions). This comprehensive study clearly demonstrates that integrating SNP, gene expression and DNA methylation data can aid in the dissection of genome regulation in a complex disease state, and Han’s methodology has the potential to be applied readily to other complex diseases.

### Inflammatory Bowel Disease

Inflammatory bowel disease (IBD) includes Ulcerative colitis (UC) and Crohn’s disease (CD), both of which are characterised by chronic inflammation in the gastrointestinal tract, are debilitating, and can lead to severe and life-threatening complications. Like MS and RA, IBD patients experience remission and relapse of symptoms. IBD is thought to arise from activation of the intestinal mucosa immune system, and the disease has been subject to extensive genetic and epigenetic examination in patients. This is largely driven *via* the collaborative effort from groups in the International IBD Genetics Consortium (Ventham et al., 2013) and currently, over 200 susceptibility loci have been identified as playing a role in IBD (Liu et al., 2015), with methylation sites in genes linked to inflammation detected in whole blood of IBD patients (Adams et al., 2014; Somineni et al., 2019). In addition to genetic risk loci, several environmental factors have been associated with the development of IBD, for example, geographic location, cigarette smoking, diet and gastrointestinal infection (Baumgart and Carding, 2007). Thus, given the complexity of IBD, an approach that integrates multi-omic data, including that of the microbiome, metabolome and proteome, will enable the identification of genomic loci that are more likely to mediate disease risk, and those which may be modified or influenced by the environment. Work in this area has begun, with a study by Ventham et al. (Ventham et al., 2016) which related genomic and gene expression data to cell methylation profile; Ventham’s study identified five differentially methylation regions and 439 differentially methylated positions that were IBD-specific, and further, by using paired genetic and epigenetic data, showed how site-specific DNA methylation changes correlate with underlying genotype differences. Therefore, since their methodology enables the relation of site-specific DNA methylation changes to underlying genotype, it provides a platform for future studies in this area, *via* the ability of this pipeline to identify biomarkers that can be used for early diagnosis and treatment.

As well as biomarkers for early diagnosis, multi-omic approaches have been used to identify patients at risk from IBD relapse. IBD is an inflammatory disease which is located in the gut, therefore many studies of the disease focus on non-genetic omics, as exemplified in a study by Borren et al. (Borren et al., 2020), who employed proteomic and metabolomic profiling of patient blood samples, with the addition of fecal metagenome sampling, to determine whether they could identify biomarkers that suggest relapse. Their data was used to generate a combined risk score of relapse from the proteomic and metabolomic profile, which was correlated with fecal microbiome composition, to indicate a correlation between particular gut microbes and risk scores, which was predictive of future risk of relapse in patients. The strength of this method is in the integration of ‘omic data from a non-invasive source (blood samples), leading to the development of a risk profile that will go some way to improving the ability to predict relapse, helping patients with the uncertainty of the future path of their condition.

The gut microbiome experiences alterations during periods of active IBD, termed functional dysbiosis, and work by Lloyd-Price and colleagues (Lloyd-Price et al., 2019) set out to understand these changes comprehensively, at the system level. As part of the Integrative Human Microbiome Project, they characterised the gut microbial ecosystem gathering multiple microbial profiles (metagenome, metatranscriptome, proteome, metabolome, and virome). Their research demonstrated characteristic changes in microbe composition, and changes to microbe transcription and metabolite pools, that were disease-specific, indicating that their integrated approach had identified relationships between multi-omic features that were potentially central to periods of IBD activity.

### Can Integrated Genomics Shed Light on the Aetiology of Multimorbidities?

Two or more NCDs often co-exist in the same individual (multimorbidity), which can further complicate the dissecting of the gene-environment interactions that are important for disease progression. Coupled with the fact that many disease-associated SNPs are in non-coding regions of the genome (Hindorff et al., 2009), and that gene regulatory elements can strongly impact distant genes strongly, limiting our capacity to make assumptions of regulatory function based on gene proximity alone (Schierding et al., 2016), a non-integrated approach that does not take into account the spatial landscape of the genome, and the interactions therein, will limit our ability to understand the aetiology of multimorbid traits. Research is currently underway to address this question, challenging our understanding of the functional impact of SNPs; specifically, Fadason et al. (Fadason et al., 2018) integrated spatial data across multiple human traits from the GWAS catalogue, identifying eQTL-eGene pairs (phenotype-associated SNP-gene pairs with confirmatory interaction data), that were missed by proximity GWAS association, as well as inter-chromosomal eQTL associations. The highlight of this approach was the ability of the research methodology to identify phenotype-associated genetic components relative to multimorbidity and individual disorders, demonstrating the strength of this approach in understanding the aetiology of complex disease, and providing a platform for the future integration of other multi-omic and environmental data.

### Current Computational and Bioinformatic Challenges/Limitations

Exploration of the highly interactive and dynamic layers of genome regulation in an integrated and informed way will enable us to begin to understand the biological basis of human diseases that are not ‘Mendelian’ (one gene, one disease) in nature. By doing so, we can improve our power to understand more about the aetiology of diseases that have a large environmental component. However, there are series of specific limitations that need to be addressed before progress can be made in this area. For example, the inclusion of additional omic data types in a study will increase the cost of that study, meaning that if budgets remain the same, sample sizes will be smaller and our power to detect associations will be reduced. Moreover, epistatic interactions are frequently ignored in analyses of genetics, and the sample sizes required to properly assess epistatic interactions are larger than those required to test single measurements against some outcome variable. This means that the addition of multiple omics further increases the complexity.

Complexity is compounded by the need for statistical power—single data sets are becoming larger as more simultaneous measurements are performed (e.g., arrays, transcriptomes, single-cell omics) and correction for multiple testing already makes discovery more difficult - combining omics increases the number of tested relationships exponentially, reducing power and increasing computational load. The difficulty is not just a matter of the availability of sufficient computational resources; development of new statistical techniques is required, and datasets must be of a sufficient size to discover underlying effects.

Thus, producing sufficiently sized datasets for multi-omic analysis will require a combination of multiple analysis runs, and either combining cohorts from multiple centres, or meta-analysis of previously produced datasets. This necessity means that dealing effectively with batch effects is highly important (Leek et al., 2010; Lazar et al., 2013; Price and Robinson, 2018). Another complication is capturing each multi-omic variable which is on its own trajectory. For instance, the timing of the process for addition of a methyl group to a cytosine will differ from the timeframe over which a difference in mRNA or protein levels are detected, or when proteins are activated by post-translational modification and localised to a particular cellular subcompartment, or the timeframe over which biological effect is observed. Analysing these events can lead to very different results purely based upon when samples are collected.

Another paradox is that DNA methylation, predominantly seen in promoter regions, is known to negatively correlate with gene expression *via* silencing of genes (Herman and Baylin, 2003). However, methylation present in the gene body is less well characterised for its involvement in gene expression (Jones, 1999). Thus, understanding this process is dependent on where events are taking place within the genome.

A major limitation currently in our investigations of the etiology of complex traits is the well-characterised bias in genomic data in public data repositories. A large proportion of genomic data is derived from populations of European ancestry, which is a major limitation given the known differences in genomic architecture between populations. This means that calculated effect sizes and risk scores based on underlying genetic variation cannot be assumed to be relevant to a global population (Martin et al., 2017; Duncan et al., 2019), with recent research clearly demonstrating the value of including diverse populations in the discovery and replication phases of GWAS, increasing the powers of discovery (Wojcik et al., 2019). The generation of diverse, globally-representative datasets for multi-omic studies is therefore a current limitation that will need to be addressed to demonstrate the applicability of techniques and research findings.

### Future Technologies

As well as improving our bioinformatics capacities to fully integrate data and environmental variables as far as possible, future strength in this area will be driven by new and emerging genome sequencing technologies, which are not yet represented in the research examples reviewed here. For example, Oxford Nanopore Technology (ONT) sequencing devices utilise nanopore channels through which DNA strands pass, each nucleotide base causing a different ionic current, which can be called. Nucleotide base calling allows for the differentiation between all five different cytosine residues (cytosine C, methylcytosine 5-mC, 5-hydroxymethlcytosine 5-hmC, formylcytosine 5-fC and 5-carboxylcytoine 5-caC) (Laszlo et al., 2013; Jain et al., 2016; Rand et al., 2017), potentially serving as a powerful tool for the integration of these data, but which is currently technically and computationally challenging on a platform that sequences all of these factors individually. A limiting factor of ONT is the currently high error rate, which has discouraged some researcher. This, however, is continuing to be addressed with the introduction of new chemistry and further optimised computational corrections (Senol Cali et al., 2019).

Rapid progress has been made in the development of single-cell omics, the profiling of single cells from a heterogenous cell population. We now have the capability to profile the genome, epigenome, transcriptome, and proteome at the single-cell level, unlike bulk sequencing, which provides comprehensive data as a single population of cells. For instance: single-cell RNA sequencing is widely applied to profile transcriptome-wide gene expression in individual cells (Tang et al., 2009); single-cell epigenomic technologies, such as chromatin immunoprecipitation sequencing (Rotem et al., 2015) and assays for transposase-accessible chromatin using sequencing (Cusanovich et al., 2015) are used to define epigenetic states of individual cells, and; several advanced approaches, including cellular indexing of transcriptomes and epitopes by sequencing (Stoeckius et al., 2017) and RNA expression and protein sequencing assay (Peterson et al., 2017) allow the simultaneous investigation of both gene and protein expression. Thus, single-cell omics is a powerful tool for elucidating cellular and microenvironmental heterogeneity in order to characterise rare cell types and explore genomic, epigenetic, transcriptomic, and proteomic regulatory mechanisms at the cellular level.

The fundamental features of single-cell omics technologies are isolating, barcoding, and sequencing individual cells to determine their DNA, mRNA, or proteins which can all be carried out in parallel. These integrative analyses allow molecules to be deciphered for genotypic and phenotypic characteristics of individual cells, and their underlying regulatory mechanisms (Chappell et al., 2018). The first and most critical step in performing single-cell omics is isolating viable single cells from a population of interest (Wang and Navin, 2015), followed by the challenge of identifying sequences from the same cells (Klein et al., 2015). Despite the technical challenges, it is now possible to define a landscape of intercellular heterogeneity and functions associated with pathophysiological processes (Lee et al., 2020). The advancement of single-cell omics and integrative analysis of the genome, epigenome, transcriptome, and proteome at the single-cell level will undoubtedly enable unprecedented levels of precision and resolution in our understanding of complex cellular systems, while also providing an unprecedented opportunity to uncover novel biological processes. Furthermore, single-cell technologies have been adapted to studies of the spatial and higher-order chromatin structure of the genome, for example, ligation- and non-ligation-based sequencing technologies [reviewed in (Zhou et al., 2021)], as well as large-scale single-cell proteomic studies [e.g., (Specht et al., 2021; Slavov, 2020)], both of which indicate the depth to which the impact of the environment on genome regulation can now be probed in a cell-specific manner. Of importance to our ability to undertake advanced multi-omic studies in single cells will be in the analysis of single-cell data, particularly as such analyses will require the generation and analysis of complex networks. Progress in this area will likely require Deep Learning and Machine Learning approaches, which are currently being developed (Ji et al., 2021) and are highlighting the use of such approaches for identifying novel biological features that has not been possible up to now. Single-cell omics will therefore be a fundamental tool in studies of the impact of the environment on genome regulation; genomic features that are modified by the environment do so in a cell- and tissue-dependent manner, meaning our ability to determine the mode of action of particular environmental variables, with respect to disease, will be greatly enhanced.

Lastly, a major consideration for our future ability to assign phenotypic impacts to environmental factors will be our power to integrate an individual’s underlying genetic propensity with the environmental risks associated with disease traits. Recent advances in the development of polygenic risk scores (PRS) for multifactorial diseases with a large degree of heritability and genetic determinants, such as most NCDs, are enabling new developments that have not been possible *via* traditional GWAS. For instance, a recent study by Hüls et al. (Hüls et al., 2021) demonstrated associations between high PRS for obesity, and sociodemographic and lifestyle factors in obese children; these associations were undetectable *via* traditional GWAS (due to the lack of power associated with large cohorts and individual loci). Further, a study of cardiovascular disease and T2D combined GWAS datasets to calculate PRS, and identified an association between high PRS and an improved disease status upon adherence to a modified lifestyle (Ye et al., 2021). This clearly highlights the potential that PRS has in capturing more of the variance of polygenic traits, and the ability of PRS to be associated with the environment. Integration of PRS may drastically improve our ability to determine the phenotypic impact of environmental factors.

## Conclusion

Integrated, multi-omic approaches, in collaboration with environmental data, will help us to robustly decipher the complex relationship between the environment, genome regulation, and associated phenotypes, to produce confirmation of the genome regulatory impacts of environmental exposures that we know are drivers of health impacts. Working in an integrated, multi-layer fashion will give us more power to predict how the environment interacts with genome regulation and influences health; however, there are challenges to develop novel statistical methods, collect cohorts of sufficient size, access sufficient computational resources to perform the analysis, and interpret the results. Future research in this area will transform our understanding of how our genomes respond to and translate an environmental exposure into a phenotype, providing new pathways for investigation into the biological basis of disease.

## References

[B1] Abarca-Gómez L. Abdeen Z. A. Hamid Z. A. Abu-Rmeileh N. M. Acosta-Cazares B. Acuin C. (2017). Worldwide Trends in Body-Mass index, Underweight, Overweight, and Obesity from 1975 to 2016: a Pooled Analysis of 2416 Population-Based Measurement Studies in 128·9 Million Children, Adolescents, and Adults. Lancet 390, 2627–2642. 10.1016/S0140-6736(17)32129-3 29029897 PMC5735219

[B2] Adams A. T. Kennedy N. A. Hansen R. Ventham N. T. OʼLeary K. R. Drummond H. E. (2014). Two-stage Genome-wide Methylation Profiling in Childhood-Onset Crohnʼs Disease Implicates Epigenetic Alterations at the VMP1/MIR21 and HLA Loci. Inflamm. Bowel Dis. 20, 1784–1793. 10.1097/mib.0000000000000179 25144570 PMC4736293

[B3] Albuquerque D. Nóbrega C. Manco L. Padez C. (2017). The Contribution of Genetics and Environment to Obesity. Br. Med. Bull. 123, 159–173. 10.1093/bmb/ldx022 28910990

[B4] Bannister A. J. Kouzarides T. (2011). Regulation of Chromatin by Histone Modifications. Cell Res 21, 381–395. 10.1038/cr.2011.22 21321607 PMC3193420

[B5] Barouki R. Gluckman P. D. Grandjean P. Hanson M. Heindel J. J. (2012). Developmental Origins of Non-communicable Disease: Implications for Research and Public Health. Environ. Health 11, 42–49. 10.1186/1476-069X-11-42 22715989 PMC3384466

[B6] Baumgart D. C. Carding S. R. (2007). Inflammatory Bowel Disease: Cause and Immunobiology. The Lancet 369, 1627–1640. 10.1016/s0140-6736(07)60750-8 17499605

[B7] Besingi W. Johansson Å. (2014). Smoke-related DNA Methylation Changes in the Etiology of Human Disease. Hum. Mol. Genet. 23, 2290–2297. 10.1093/hmg/ddt621 24334605

[B8] Bickmore W. A. van Steensel B. (2013). Genome Architecture: Domain Organization of Interphase Chromosomes. Cell 152, 1270–1284. 10.1016/j.cell.2013.02.001 23498936

[B9] Bjornevik K. Cortese M. Healy B. C. Kuhle J. Mina M. J. Leng Y. (2022). Longitudinal Analysis Reveals High Prevalence of Epstein-Barr Virus Associated with Multiple Sclerosis. Science 375 (6578), 296–301. 10.1126/science.abj8222 35025605

[B10] Bonev B. Cavalli G. (2016). Organization and Function of the 3D Genome. Nat. Rev. Genet. 17, 661–678. 10.1038/nrg.2016.112 27739532

[B11] Borren N. Z. Plichta D. Joshi A. D. Bonilla G. Sadreyev R. Vlamakis H. (2020). Multi-"-Omics" Profiling in Patients with Quiescent Inflammatory Bowel Disease Identifies Biomarkers Predicting Relapse. Inflamm. Bowel Dis. 26, 1524–1532. 10.1093/ibd/izaa183 32766830 PMC7500522

[B12] Buenrostro J. D. Wu B. Chang H. Y. Greenleaf W. J. (2015). ATAC‐seq: a Method for Assaying Chromatin Accessibility Genome‐wide. Curr. Protoc. Mol. Biol. 109, 21.29. 1–21.29. 9. 10.1002/0471142727.mb2129s109 PMC4374986 25559105

[B13] Calamini B. Morimoto R. I. (2012). Protein Homeostasis as a Therapeutic Target for Diseases of Protein Conformation. Curr. Top. Med. Chem. 12, 2623–2640. 10.2174/1568026611212220014 23339312 PMC3955168

[B14] Castel S. E. Martienssen R. A. (2013). RNA Interference in the Nucleus: Roles for Small RNAs in Transcription, Epigenetics and beyond. Nat. Rev. Genet. 14, 100–112. 10.1038/nrg3355 23329111 PMC4205957

[B15] Cervantes-Gracia K. Husi H. (2018). Integrative Analysis of Multiple Sclerosis Using a Systems Biology Approach. Sci. Rep. 8, 5633. 10.1038/s41598-018-24032-8 29618802 PMC5884799

[B16] Chappell L. Russell A. J. C. Voet T. (2018). Single-Cell (Multi)omics Technologies. Annu. Rev. Genom. Hum. Genet. 19, 15–41. 10.1146/annurev-genom-091416-035324 29727584

[B17] Chen N. Miao L. Lin W. Zou D. Huang L. Huang J. (2021). Integrated DNA Methylation and Gene Expression Analysis Identified S100A8 and S100A9 in the Pathogenesis of Obesity. Front. Cardiovasc. Med. 8, 350. 10.3389/fcvm.2021.631650 PMC8163519 34055926

[B18] Consortium I. M. S. G. Hafler D. A. Compston A. Sawcer S. Lander E. S. Daly M. J. (2007). Risk Alleles for Multiple Sclerosis Identified by a Genomewide Study. N. Engl. J. Med. 357, 851–862. 10.1056/NEJMoa073493 17660530

[B19] Corbett S. Courtiol A. Lummaa V. Moorad J. Stearns S. (2018). The Transition to Modernity and Chronic Disease: Mismatch and Natural Selection. Nat. Rev. Genet. 19, 419–430. 10.1038/s41576-018-0012-3 29743650

[B20] Cottrell E. C. Ozanne S. E. (2008). Early Life Programming of Obesity and Metabolic Disease. Physiol. Behav. 94, 17–28. 10.1016/j.physbeh.2007.11.017 18155097

[B21] Cremer T. Cremer C. (2001). Chromosome Territories, Nuclear Architecture and Gene Regulation in Mammalian Cells. Nat. Rev. Genet. 2, 292–301. 10.1038/35066075 11283701

[B22] Cubeñas-Potts C. Corces V. G. (2015). Architectural Proteins, Transcription, and the Three‐dimensional Organization of the Genome. FEBS Lett. 589, 2923–2930. 26008126 10.1016/j.febslet.2015.05.025 PMC4598269

[B23] Cusanovich D. A. Daza R. Adey A. Pliner H. A. Christiansen L. Gunderson K. L. (2015). Multiplex Single-Cell Profiling of Chromatin Accessibility by Combinatorial Cellular Indexing. Science 348, 910–914. 10.1126/science.aab1601 25953818 PMC4836442

[B24] Dai H. Wang Z. (2014). Histone Modification Patterns and Their Responses to Environment. Curr. Envir Health Rpt 1, 11–21. 10.1007/s40572-013-0008-2

[B25] De Jager P. L. Ma Y. McCabe C. Xu J. Vardarajan B. N. Felsky D. (2018). A Multi-Omic Atlas of the Human Frontal Cortex for Aging and Alzheimer's Disease Research. Sci. Data 5, 180142. 10.1038/sdata.2018.142 30084846 PMC6080491

[B26] De Nadal E. Ammerer G. Posas F. (2011). Controlling Gene Expression in Response to Stress. Nat. Rev. Genet. 12, 833–845. 10.1038/nrg3055 22048664

[B27] Deane K. D. Demoruelle M. K. Kelmenson L. B. Kuhn K. A. Norris J. M. Holers V. M. (2017). Genetic and Environmental Risk Factors for Rheumatoid Arthritis. Best Pract. Res. Clin. Rheumatol. 31, 3–18. 10.1016/j.berh.2017.08.003 29221595 PMC5726551

[B28] Dekker J. Misteli T. (2015). Long-range Chromatin Interactions. Cold Spring Harb Perspect. Biol. 7, a019356. 10.1101/cshperspect.a019356 26430217 PMC4588061

[B29] Dixon J. R. Selvaraj S. Yue F. Kim A. Li Y. Shen Y. (2012). Topological Domains in Mammalian Genomes Identified by Analysis of Chromatin Interactions. Nature 485, 376–380. 10.1038/nature11082 22495300 PMC3356448

[B30] Duncan L. Shen H. Gelaye B. Meijsen J. Ressler K. Feldman M. (2019). Analysis of Polygenic Risk Score Usage and Performance in Diverse Human Populations. Nat. Commun. 10, 3328–3329. 10.1038/s41467-019-11112-0 31346163 PMC6658471

[B31] Engreitz J. M. Ollikainen N. Guttman M. (2016). Long Non-coding RNAs: Spatial Amplifiers that Control Nuclear Structure and Gene Expression. Nat. Rev. Mol. Cel Biol 17, 756–770. 10.1038/nrm.2016.126 27780979

[B32] Esteller M. (2007). Cancer Epigenomics: DNA Methylomes and Histone-Modification Maps. Nat. Rev. Genet. 8, 286–298. 10.1038/nrg2005 17339880

[B33] Fadason T. Ekblad C. Ingram J. R. Schierding W. S. O'Sullivan J. M. (2017). Physical Interactions and Expression Quantitative Traits Loci Identify Regulatory Connections for Obesity and Type 2 Diabetes Associated SNPs. Front. Genet. 8, 150. 10.3389/fgene.2017.00150 29081791 PMC5645506

[B34] Fadason T. Schierding W. Lumley T. O'Sullivan J. M. (2018). Chromatin Interactions and Expression Quantitative Trait Loci Reveal Genetic Drivers of Multimorbidities. Nat. Commun. 9, 5198. 10.1038/s41467-018-07692-y 30518762 PMC6281603

[B35] Fang L. Wuptra K. Chen D. Li H. Huang S. K. Jin C. (2014). Environmental-stress-induced Chromatin Regulation and its Heritability. J. Carcinog Mutagen 5. 10.4172/2157-2518.1000156 PMC4101908 25045581

[B36] Feil R. Fraga M. F. (2012). Epigenetics and the Environment: Emerging Patterns and Implications. Nat. Rev. Genet. 13, 97–109. 10.1038/nrg3142 22215131

[B37] Fernández-Tajes J. Gaulton K. J. van de Bunt M. Torres J. Thurner M. Mahajan A. (2019). Developing a Network View of Type 2 Diabetes Risk Pathways through Integration of Genetic, Genomic and Functional Data. Genome Med. 11, 1–14. 30914061 10.1186/s13073-019-0628-8 PMC6436236

[B38] Fiehn O. Garvey W. T. Newman J. W. Lok K. H. Hoppel C. L. Adams S. H. (2010). Plasma Metabolomic Profiles Reflective of Glucose Homeostasis in Non-diabetic and Type 2 Diabetic Obese African-American Women. PloS one 5, e15234. 10.1371/journal.pone.0015234 21170321 PMC3000813

[B39] Florath I. Butterbach K. Muller H. Bewerunge-Hudler M. Brenner H. (2014). Cross-sectional and Longitudinal Changes in DNA Methylation with Age: an Epigenome-wide Analysis Revealing over 60 Novel Age-Associated CpG Sites. Hum. Mol. Genet. 23, 1186–1201. 10.1093/hmg/ddt531 24163245 PMC3919014

[B40] Franke M. Ibrahim D. M. Andrey G. Schwarzer W. Heinrich V. Schöpflin R. (2016). Formation of New Chromatin Domains Determines Pathogenicity of Genomic Duplications. Nature 538, 265–269. 10.1038/nature19800 27706140

[B41] Freeman J. R. Chu S. Hsu T. Huang Y.-T. (2016). Epigenome-wide Association Study of Smoking and DNA Methylation in Non-small Cell Lung Neoplasms. Oncotarget 7, 69579–69591. 10.18632/oncotarget.11831 27602958 PMC5342499

[B42] Garrison C. B. Lastwika K. J. Zhang Y. Li C. I. Lampe P. D. (2017). Proteomic Analysis, Immune Dysregulation, and Pathway Interconnections with Obesity. J. Proteome Res. 16, 274–287. 10.1021/acs.jproteome.6b00611 27769113 PMC5234688

[B43] Ghazalpour A. Bennett B. Petyuk V. A. Orozco L. Hagopian R. Mungrue I. N. (2011). Comparative Analysis of Proteome and Transcriptome Variation in Mouse. Plos Genet. 7, e1001393. 10.1371/journal.pgen.1001393 21695224 PMC3111477

[B44] Gluckman P. D. Hanson M. A. Cooper C. Thornburg K. L. (2008). Effect of In Utero and Early-Life Conditions on Adult Health and Disease. N. Engl. J. Med. 359, 61–73. 10.1056/nejmra0708473 18596274 PMC3923653

[B45] Gokuladhas S. Schierding W. Cameron-Smith D. Wake M. Scotter E. L. O’Sullivan J. (2020). Shared Regulatory Pathways Reveal Novel Genetic Correlations between Grip Strength and Neuromuscular Disorders. Front. Genet. 11, 393. 10.3389/fgene.2020.00393 32391060 PMC7194178

[B46] Gokuladhas S. Schierding W. Golovina E. Fadason T. O'Sullivan J. M. (2021). Unravelling the Shared Genetic Mechanisms Underlying 18 Autoimmune Diseases Using a Systems Approach. Front. Immunol. 10.3389/fimmu.2021.693142 PMC8415031 34484189

[B47] Guttman M. Rinn J. L. (2012). Modular Regulatory Principles of Large Non-coding RNAs. Nature 482, 339–346. 10.1038/nature10887 22337053 PMC4197003

[B48] Ha E. Bang S.-Y. Lim J. Yun J. H. Kim J.-M. Bae J.-B. (2021). Genetic Variants Shape Rheumatoid Arthritis-specific Transcriptomic Features in CD4+ T Cells through Differential DNA Methylation, Explaining a Substantial Proportion of Heritability. Ann. Rheum. Dis. 80, 876–883. 10.1136/annrheumdis-2020-219152 33436383

[B49] Hartstra A. V. Bouter K. E. C. Bäckhed F. Nieuwdorp M. (2015). Insights into the Role of the Microbiome in Obesity and Type 2 Diabetes. Diabetes care 38, 159–165. 10.2337/dc14-0769 25538312

[B50] Herman J. G. Baylin S. B. (2003). Gene Silencing in Cancer in Association with Promoter Hypermethylation. N. Engl. J. Med. 349, 2042–2054. 10.1056/nejmra023075 14627790

[B51] Hindorff L. A. Sethupathy P. Junkins H. A. Ramos E. M. Mehta J. P. Collins F. S. (2009). Potential Etiologic and Functional Implications of Genome-wide Association Loci for Human Diseases and Traits. Proc. Natl. Acad. Sci. 106, 9362–9367. 10.1073/pnas.0903103106 19474294 PMC2687147

[B52] Hosseini A. Babaloo Z. Gharibi T. Shomali N. Marofi F. Hashemi V. (2020). Epigenetic Mechanisms Shape the Underlining Expression Regulatory Mechanisms of the STAT3 in Multiple Sclerosis Disease. BMC Res. Notes 13, 568. 10.1186/s13104-020-05427-1 33375941 PMC7771087

[B53] Hüls A. Wright M. N. Bogl L. H. Kaprio J. Lissner L. Molnár D. (2021). Polygenic Risk for Obesity and its Interaction with Lifestyle and Sociodemographic Factors in European Children and Adolescents. Int. J. Obes. 45, 1321–1330. 10.1038/s41366-021-00795-5 PMC8159747 33753884

[B54] International Multiple Sclerosis Genetics Consortium (2019). Multiple Sclerosis Genomic Map Implicates Peripheral Immune Cells and Microglia in Susceptibility. Science 365, eaav7188. 10.1126/science.aav7188 31604244 PMC7241648

[B55] Ivorra C. Fraga M. F. Bayón G. F. Fernández A. F. Garcia-Vicent C. Chaves F. J. (2015). DNA Methylation Patterns in Newborns Exposed to Tobacco In Utero. J. Transl Med. 13, 25–29. 10.1186/s12967-015-0384-5 25623364 PMC4312439

[B56] Jacobs B. M. Noyce A. J. Bestwick J. Belete D. Giovannoni G. Dobson R. (2021). Gene-Environment Interactions in Multiple Sclerosis: A UK Biobank Study. Neurology-Neuroimmunology Neuroinflammation 8. 10.1212/nxi.0000000000001007 PMC8192056 34049995

[B57] Jaenisch R. Bird A. (2003). Epigenetic Regulation of Gene Expression: How the Genome Integrates Intrinsic and Environmental Signals. Nat. Genet. 33, 245–254. 10.1038/ng1089 12610534

[B58] Jaffe A. E. Irizarry R. A. (2014). Accounting for Cellular Heterogeneity Is Critical in Epigenome-wide Association Studies. Genome Biol. 15, R31–R39. 10.1186/gb-2014-15-2-r31 24495553 PMC4053810

[B59] Jain M. Olsen H. E. Paten B. Akeson M. (2016). Erratum to: The Oxford Nanopore MinION: Delivery of Nanopore Sequencing to the Genomics Community. Genome Biol. 17, 239. 10.1186/s13059-016-1103-0 27887629 PMC5124260

[B60] Jayasinghe T. N. Chiavaroli V. Holland D. J. Cutfield W. S. O'Sullivan J. M. (2016). The new era of Treatment for Obesity and Metabolic Disorders: Evidence and Expectations for Gut Microbiome Transplantation. Front. Cel. Infect. Microbiol. 6, 15. 10.3389/fcimb.2016.00015 PMC4759265 26925392

[B61] Ji Y. Lotfollahi M. Wolf F. A. Theis F. J. (2021). Machine Learning for Perturbational Single-Cell Omics. Cel Syst. 12, 522–537. 10.1016/j.cels.2021.05.016 34139164

[B62] Jirtle R. L. Skinner M. K. (2007). Environmental Epigenomics and Disease Susceptibility. Nat. Rev. Genet. 8, 253–262. 10.1038/nrg2045 17363974 PMC5940010

[B63] Johnson W. Li L. Kuh D. Hardy R. (2015). How Has the Age-Related Process of Overweight or Obesity Development Changed over Time? Co-ordinated Analyses of Individual Participant Data from Five United Kingdom Birth Cohorts. Plos Med. 12, e1001828. discussion e1001828. 10.1371/journal.pmed.1001828 25993005 PMC4437909

[B64] Jones P. A. (1999). The DNA Methylation Paradox. Trends Genetics 15, 34–37. 10.1016/s0168-9525(98)01636-9 10087932

[B65] Joslin A. C. Sobreira D. R. Hansen G. T. Sakabe N. J. Aneas I. Montefiori L. E. (2021). A Functional Genomics Pipeline Identifies Pleiotropy and Cross-Tissue Effects within Obesity-Associated GWAS Loci. Nat. Commun. 12, 1–15. 10.1038/s41467-021-25614-3 34489471 PMC8421397

[B66] Joubert B. R. Håberg S. E. Nilsen R. M. Wang X. Vollset S. E. Murphy S. K. (2012). 450K Epigenome-wide Scan Identifies Differential DNA Methylation in Newborns Related to Maternal Smoking during Pregnancy. Environ. Health Perspect. 120, 1425–1431. 10.1289/ehp.1205412 22851337 PMC3491949

[B67] Kaiser A. B. Zhang N. Van Der Pluijm W. (2018). Global Prevalence of Type 2 Diabetes over the Next Ten Years (2018-2028). Diabetes 67 (Supplement_1), 202-LB. 10.2337/db18-202-LB

[B68] Khawandanah J. (2019). Double or Hybrid Diabetes: A Systematic Review on Disease Prevalence, Characteristics and Risk Factors. Nutr. Diabetes 9, 33–39. 10.1038/s41387-019-0101-1 31685799 PMC6828774

[B69] Klein A. M. Mazutis L. Akartuna I. Tallapragada N. Veres A. Li V. (2015). Droplet Barcoding for Single-Cell Transcriptomics Applied to Embryonic Stem Cells. Cell 161, 1187–1201. 10.1016/j.cell.2015.04.044 26000487 PMC4441768

[B70] Koga H. Kaushik S. Cuervo A. M. (2011). Protein Homeostasis and Aging: The Importance of Exquisite Quality Control. Ageing Res. Rev. 10, 205–215. 10.1016/j.arr.2010.02.001 20152936 PMC2888802

[B71] Krijger P. H. L. De Laat W. (2016). Regulation of Disease-Associated Gene Expression in the 3D Genome. Nat. Rev. Mol. Cel Biol 17, 771–782. 10.1038/nrm.2016.138 27826147

[B72] Kurilshikov A. van den Munckhof I. C. L. Chen L. Bonder M. J. Schraa K. Rutten J. H. W. (2019). Gut Microbial Associations to Plasma Metabolites Linked to Cardiovascular Phenotypes and Risk. Circ. Res. 124, 1808–1820. 10.1161/circresaha.118.314642 30971183

[B73] La Cognata V. Morello G. Cavallaro S. (2021). Omics Data and Their Integrative Analysis to Support Stratified Medicine in Neurodegenerative Diseases. Ijms 22, 4820. 10.3390/ijms22094820 34062930 PMC8125201

[B74] Laszlo A. H. Derrington I. M. Brinkerhoff H. Langford K. W. Nova I. C. Samson J. M. (2013). Detection and Mapping of 5-methylcytosine and 5-hydroxymethylcytosine with Nanopore MspA. Proc. Natl. Acad. Sci. 110, 18904–18909. 10.1073/pnas.1310240110 24167255 PMC3839702

[B75] Lazar C. Meganck S. Taminau J. Steenhoff D. Coletta A. Molter C. (2013). Batch Effect Removal Methods for Microarray Gene Expression Data Integration: a Survey. Brief. Bioinformatics 14, 469–490. 10.1093/bib/bbs037 22851511

[B76] Lee Y. M. Li C. Lam S. H. Gong Z. Lin Q. (2018). Proteomic Analysis of Zebrafish (*Danio rerio*) after Chemical Exposure. Springer 1797, 443–459. 10.1007/978-1-4939-7883-0_24 29896708

[B77] Lee J. Hyeon D. Y. Hwang D. (2020). Single-cell Multiomics: Technologies and Data Analysis Methods. Exp. Mol. Med. 52, 1428–1442. 10.1038/s12276-020-0420-2 32929225 PMC8080692

[B78] Leek J. T. Scharpf R. B. Bravo H. C. Simcha D. Langmead B. Johnson W. E. (2010). Tackling the Widespread and Critical Impact of Batch Effects in High-Throughput Data. Nat. Rev. Genet. 11, 733–739. 10.1038/nrg2825 20838408 PMC3880143

[B79] Li Yim A. Y. Ferrero E. Maratou K. Lewis H. D. Royal G. Tough D. F. (2021). Novel Insights into Rheumatoid Arthritis through Characterization of Concordant Changes in DNA Methylation and Gene Expression in Synovial Biopsies of Patients with Differing Numbers of Swollen Joints. Front. Immunol. 12, 1215. 10.3389/fimmu.2021.651475 PMC8100206 33968050

[B80] Lieberman-Aiden E. Van Berkum N. L. Williams L. Imakaev M. Ragoczy T. Telling A. (2009). Comprehensive Mapping of Long-Range Interactions Reveals Folding Principles of the Human Genome. science 326, 289–293. 10.1126/science.1181369 19815776 PMC2858594

[B81] Lillycrop K. A. Burdge G. C. (2012). Epigenetic Mechanisms Linking Early Nutrition to Long Term Health. Best Pract. Res. Clin. Endocrinol. Metab. 26, 667–676. 10.1016/j.beem.2012.03.009 22980048

[B82] Liu J. Z. Van Sommeren S. van Sommeren S. Huang H. Ng S. C. Alberts R. (2015). Association Analyses Identify 38 Susceptibility Loci for Inflammatory Bowel Disease and Highlight Shared Genetic Risk across Populations. Nat. Genet. 47, 979–986. 10.1038/ng.3359 26192919 PMC4881818

[B83] Lloyd-Price J. Arze C. Arze C. Ananthakrishnan A. N. Schirmer M. Avila-Pacheco J. (2019). Multi-omics of the Gut Microbial Ecosystem in Inflammatory Bowel Diseases. Nature 569, 655–662. 10.1038/s41586-019-1237-9 31142855 PMC6650278

[B84] Lujambio A. Portela A. Liz J. Melo S. A. Rossi S. Spizzo R. (2010). CpG Island Hypermethylation-Associated Silencing of Non-coding RNAs Transcribed from Ultraconserved Regions in Human Cancer. Oncogene 29, 6390–6401. 10.1038/onc.2010.361 20802525 PMC3007676

[B85] Manolio T. A. Collins F. S. Cox N. J. Goldstein D. B. Hindorff L. A. Hunter D. J. (2009). Finding the Missing Heritability of Complex Diseases. Nature 461, 747–753. 10.1038/nature08494 19812666 PMC2831613

[B86] Marchesi J. R. Ravel J. (2015). The Vocabulary of Microbiome Research: A Proposal. Springer. 10.1186/s40168-015-0094-5 PMC4520061 26229597

[B87] Martin A. R. Gignoux C. R. Walters R. K. Wojcik G. L. Neale B. M. Gravel S. (2017). Human Demographic History Impacts Genetic Risk Prediction across Diverse Populations. Am. J. Hum. Genet. 100, 635–649. 10.1016/j.ajhg.2017.03.004 28366442 PMC5384097

[B88] Maruvada P. Leone V. Kaplan L. M. Chang E. B. (2017). The Human Microbiome and Obesity: Moving beyond Associations. Cell Host & Microbe 22, 589–599. 10.1016/j.chom.2017.10.005 29120742

[B89] Matilainen O. Sleiman M. S. B. Quiros P. M. Garcia S. M. D. A. Auwerx J. (2017). The Chromatin Remodeling Factor ISW-1 Integrates Organismal Responses against Nuclear and Mitochondrial Stress. Nat. Commun. 8, 1818. 10.1038/s41467-017-01903-8 29180639 PMC5703887

[B90] Mehta D. Jackson R. Paul G. Shi J. Sabbagh M. (2017). Why Do Trials for Alzheimer's Disease Drugs Keep Failing? A Discontinued Drug Perspective for 2010-2015. Expert Opin. Investig. Drugs 26, 735–739. 10.1080/13543784.2017.1323868 PMC5576861 28460541

[B91] Mens M. M. J. Maas S. C. E. Klap J. Weverling G. J. Klatser P. Brakenhoff J. P. J. (2020). Multi-omics Analysis Reveals microRNAs Associated with Cardiometabolic Traits. Front. Genet. 11, 110. 10.3389/fgene.2020.00110 32174972 PMC7056871

[B92] Merino J. Leong A. Liu C.-T. Porneala B. Walford G. A. von Grotthuss M. (2018). Metabolomics Insights into Early Type 2 Diabetes Pathogenesis and Detection in Individuals with normal Fasting Glucose. Diabetologia 61, 1315–1324. 10.1007/s00125-018-4599-x 29626220 PMC5940516

[B93] Mo X.-B. Lei S.-F. Qian Q.-Y. Guo Y.-F. Zhang Y.-H. Zhang H. (2019). Integrative Analysis Revealed Potential Causal Genetic and Epigenetic Factors for Multiple Sclerosis. J. Neurol. 266, 2699–2709. 10.1007/s00415-019-09476-w 31321514

[B94] Nativio R. Lan Y. Donahue G. Sidoli S. Berson A. Srinivasan A. R. (2020). An Integrated Multi-Omics Approach Identifies Epigenetic Alterations Associated with Alzheimer's Disease. Nat. Genet. 52, 1024–1035. 10.1038/s41588-020-0696-0 32989324 PMC8098004

[B95] Neff R. A. Wang M. Vatansever S. Guo L. Ming C. Wang Q. (2021). Molecular Subtyping of Alzheimer’s Disease Using RNA Sequencing Data Reveals Novel Mechanisms and Targets. Sci. Adv. 7, eabb5398. 10.1126/sciadv.abb5398 33523961 PMC7787497

[B96] Noble A. J. Pearson J. F. Noble A. D. Boden J. M. Horwood L. J. Kennedy M. A. (2021). DNA Methylation Analysis Using Bisulfite-Based Amplicon Sequencing of Individuals Exposed to Maternal Tobacco Use during Pregnancy, and Offspring Conduct Problems in Childhood and Adolescence. Reproduction. Fertility and Development In Press. 10.1071/RD21108 35412968

[B97] Noce A. Marrone G. Di Daniele F. Ottaviani E. Wilson Jones G. Bernini R. (2019). Impact of Gut Microbiota Composition on Onset and Progression of Chronic Non-communicable Diseases. Nutrients 11, 1073. 10.3390/nu11051073 PMC6567014 31091761

[B98] Nora E. P. Lajoie B. R. Schulz E. G. Giorgetti L. Okamoto I. Servant N. (2012). Spatial Partitioning of the Regulatory Landscape of the X-Inactivation centre. Nature 485, 381–385. 10.1038/nature11049 22495304 PMC3555144

[B99] Nyaga D. M. Vickers M. H. Jefferies C. Fadason T. O’Sullivan J. M. (2021). Untangling the Genetic Link between Type 1 and Type 2 Diabetes Using Functional Genomics. Scientific Rep. 11, 1–15. 10.1038/s41598-021-93346-x PMC8260770 34230558

[B100] O’Gorman C. Lucas R. Taylor B. (2012). Environmental Risk Factors for Multiple Sclerosis: a Review with a Focus on Molecular Mechanisms. Int. J. Mol. Sci. 13, 11718–11752. 23109880 10.3390/ijms130911718 PMC3472772

[B101] Østergaard S. D. Mukherjee S. Sharp S. J. Proitsi P. Lotta L. A. Day F. (2015). Associations between Potentially Modifiable Risk Factors and Alzheimer Disease: a Mendelian Randomization Study. PLoS Med. 12, e1001841. 26079503 10.1371/journal.pmed.1001841 PMC4469461

[B102] Palmqvist S. Janelidze S. Quiroz Y. T. Zetterberg H. Lopera F. Stomrud E. (2020). Discriminative Accuracy of Plasma Phospho-Tau217 for Alzheimer Disease vs Other Neurodegenerative Disorders. Jama 324, 772–781. 10.1001/jama.2020.12134 32722745 PMC7388060

[B103] Peters L. Meister G. (2007). Argonaute Proteins: Mediators of RNA Silencing. Mol. Cel. 26, 611–623. 10.1016/j.molcel.2007.05.001 17560368

[B104] Peterson V. M. Zhang K. X. Kumar N. Wong J. Li L. Wilson D. C. (2017). Multiplexed Quantification of Proteins and Transcripts in Single Cells. Nat. Biotechnol. 35, 936–939. 10.1038/nbt.3973 28854175

[B105] Petesch S. J. Lis J. T. (2008). Rapid, Transcription-independent Loss of Nucleosomes over a Large Chromatin Domain at Hsp70 Loci. Cell 134, 74–84. 10.1016/j.cell.2008.05.029 18614012 PMC2527511

[B106] Pingault J.-B. O’Reilly P. F. Schoeler T. Ploubidis G. B. Rijsdijk F. Dudbridge F. (2018). Using Genetic Data to Strengthen Causal Inference in Observational Research. Nat. Rev. Genet. 19, 566–580. 10.1038/s41576-018-0020-3 29872216

[B107] Price E. M. Robinson W. P. (2018). Adjusting for Batch Effects in DNA Methylation Microarray Data, a Lesson Learned. Front. Genet. 9, 83. 10.3389/fgene.2018.00083 29616078 PMC5864890

[B108] Rand A. C. Jain M. Eizenga J. M. Musselman-Brown A. Olsen H. E. Akeson M. (2017). Mapping DNA Methylation with High-Throughput Nanopore Sequencing. Nat. Methods 14, 411–413. 10.1038/nmeth.4189 28218897 PMC5704956

[B109] Rauschert S. Kirchberg F. F. Marchioro L. Koletzko B. Hellmuth C. Uhl O. (2017). Early Programming of Obesity throughout the Life Course: a Metabolomics Perspective. Ann. Nutr. Metab. 70, 201–209. 10.1159/000459635 28301839

[B110] Raychaudhuri S. Sandor C. Stahl E. A. Freudenberg J. Lee H.-S. Jia X. (2012). Five Amino Acids in Three HLA Proteins Explain Most of the Association between MHC and Seropositive Rheumatoid Arthritis. Nat. Genet. 44, 291–296. 10.1038/ng.1076 22286218 PMC3288335

[B111] Risca V. I. Greenleaf W. J. (2015). Unraveling the 3D Genome: Genomics Tools for Multiscale Exploration. Trends Genet. 31, 357–372. 10.1016/j.tig.2015.03.010 25887733 PMC4490074

[B112] Rotem A. Ram O. Shoresh N. Sperling R. A. Goren A. Weitz D. A. (2015). Single-cell ChIP-Seq Reveals Cell Subpopulations Defined by Chromatin State. Nat. Biotechnol. 33, 1165–1172. 10.1038/nbt.3383 26458175 PMC4636926

[B113] Rowley M. J. Corces V. G. (2016). The Three-Dimensional Genome: Principles and Roles of Long-Distance Interactions. Curr. Opin. Cel Biol. 40, 8–14. 10.1016/j.ceb.2016.01.009 PMC4887315 26852111

[B114] Sarli P.-M. Manousopoulou A. Efthymiou E. Zouridis A. Potiris A. Pervanidou P. (2021). Liver Proteome Profile of Growth Restricted and Appropriately Grown Newborn Wistar Rats Associated with Maternal Undernutrition. Front. Endocrinol. 12. 10.3389/fendo.2021.684220 PMC8195994 34127923

[B115] Saxena A. Carninci P. (2011). Long Non-coding RNA Modifies Chromatin. Bioessays 33, 830–839. 10.1002/bies.201100084 21915889 PMC3258546

[B116] Sayols-Baixeras S. Subirana I. Fernández-Sanlés A. Sentí M. Lluís-Ganella C. Marrugat J. (2017). DNA Methylation and Obesity Traits: An Epigenome-wide Association Study. The REGICOR Study. Epigenetics 12, 909–916. 10.1080/15592294.2017.1363951 29099282 PMC5788444

[B117] Schierding W. O’Sullivan J. M. (2015). Connecting SNPs in Diabetes: a Spatial Analysis of Meta-GWAS Loci. Front. Endocrinol. 6, 102. 10.3389/fendo.2015.00102 PMC4490250 26191039

[B118] Schierding W. Antony J. Cutfield W. S. Horsfield J. A. O’Sullivan J. M. (2016). Intergenic GWAS SNPs Are Key Components of the Spatial and Regulatory Network for Human Growth. Hum. Mol. Genet. 25, 3372–3382. 10.1093/hmg/ddw165 27288450

[B119] Senol Cali D. Kim J. S. Ghose S. Alkan C. Mutlu O. (2019). Nanopore Sequencing Technology and Tools for Genome Assembly: Computational Analysis of the Current State, Bottlenecks and Future Directions. Brief. Bioinformatics 20, 1542–1559. 10.1093/bib/bby017 29617724 PMC6781587

[B120] Sexton T. Yaffe E. Kenigsberg E. Bantignies F. Leblanc B. Hoichman M. (2012). Three-dimensional Folding and Functional Organization Principles of the Drosophila Genome. Cell 148, 458–472. 10.1016/j.cell.2012.01.010 22265598

[B121] Silventoinen K. Jelenkovic A. Sund R. Hur Y.-M. Yokoyama Y. Honda C. (2016). Genetic and Environmental Effects on Body Mass index from Infancy to the Onset of Adulthood: an Individual-Based Pooled Analysis of 45 Twin Cohorts Participating in the COllaborative Project of Development of Anthropometrical Measures in Twins (CODATwins) Study. Am. J. Clin. Nutr. 104, 371–379. 10.3945/ajcn.116.130252 27413137 PMC4962159

[B122] Sinha I. Modesto J. Krebs N. M. Stanley A. E. Walter V. A. Richie J. P. Jr (2021). Changes in Salivary Proteome before and after Cigarette Smoking in Smokers Compared to Sham Smoking in Nonsmokers: A Pilot Study. Tob. Induced Dis. 19. 10.18332/tid/138336 PMC8240953 34239408

[B123] Slavov N. (2020). Unpicking the Proteome in Single Cells. Science 367, 512–513. 10.1126/science.aaz6695 32001644 PMC7029782

[B124] Somineni H. K. Venkateswaran S. Kilaru V. Marigorta U. M. Mo A. Okou D. T. (2019). Blood-derived DNA Methylation Signatures of Crohn's Disease and Severity of Intestinal Inflammation. Gastroenterology 156, 2254–2265. e3. 10.1053/j.gastro.2019.01.270 30779925 PMC6529254

[B125] Specht H. Emmott E. Petelski A. A. Huffman R. G. Perlman D. H. Serra M. (2021). Single-cell Proteomic and Transcriptomic Analysis of Macrophage Heterogeneity Using SCoPE2. Genome Biol. 22, 50–27. 10.1186/s13059-021-02267-5 33504367 PMC7839219

[B126] Stevens T. J. Lando D. Basu S. Atkinson L. P. Cao Y. Lee S. F. (2017). 3D Structures of Individual Mammalian Genomes Studied by Single-Cell Hi-C. Nature 544, 59–64. 10.1038/nature21429 28289288 PMC5385134

[B127] Stoeckius M. Hafemeister C. Stephenson W. Houck-Loomis B. Chattopadhyay P. K. Swerdlow H. (2017). Simultaneous Epitope and Transcriptome Measurement in Single Cells. Nat. Methods 14, 865–868. 10.1038/nmeth.4380 28759029 PMC5669064

[B128] Tang F. Barbacioru C. Wang Y. Nordman E. Lee C. Xu N. (2009). mRNA-Seq Whole-Transcriptome Analysis of a Single Cell. Nat. Methods 6, 377–382. 10.1038/nmeth.1315 19349980

[B129] Tani H. Onuma Y. Ito Y. Torimura M. (2014). Long Non-coding RNAs as Surrogate Indicators for Chemical Stress Responses in Human-Induced Pluripotent Stem Cells. PloS one 9, e106282. 10.1371/journal.pone.0106282 25171338 PMC4149554

[B130] Timpson N. J. Lawlor D. A. Harbord R. M. Gaunt T. R. Day I. N. Palmer L. J. (2005). C-reactive Protein and its Role in Metabolic Syndrome: Mendelian Randomisation Study. The Lancet 366, 1954–1959. 10.1016/s0140-6736(05)67786-0 16325697

[B131] Tounian P. (2011). Programming towards Childhood Obesity. Ann. Nutr. Metab. 58, 30–41. 10.1159/000328038 21846979

[B132] Tsai Y.-W. Dong J.-L. Jian Y.-J. Fu S.-H. Chien M.-W. Liu Y.-W. (2021). Gut Microbiota-Modulated Metabolomic Profiling Shapes the Etiology and Pathogenesis of Autoimmune Diseases. Microorganisms 9, 1930. 10.3390/microorganisms9091930 34576825 PMC8466726

[B133] Vaissière T. Sawan C. Herceg Z. (2008). Epigenetic Interplay between Histone Modifications and DNA Methylation in Gene Silencing. Mutat. Research/Reviews Mutat. Res. 659, 40–48. 18407786 10.1016/j.mrrev.2008.02.004

[B134] Van Cauwenberghe C. Van Broeckhoven C. Sleegers K. (2016). The Genetic Landscape of Alzheimer Disease: Clinical Implications and Perspectives. Genet. Med. 18, 421–430. 10.1038/gim.2015.117 26312828 PMC4857183

[B135] van der Kolk B. W. Saari S. Lovric A. Arif M. Alvarez M. Ko A. (2021). Molecular Pathways behind Acquired Obesity: Adipose Tissue and Skeletal Muscle Multiomics in Monozygotic Twin Pairs Discordant for BMI. Cel Rep. Med. 2, 100226. 10.1016/j.xcrm.2021.100226 PMC8080113 33948567

[B136] Ventham N. T. Kennedy N. A. Nimmo E. R. Satsangi J. (2013). Beyond Gene Discovery in Inflammatory Bowel Disease: the Emerging Role of Epigenetics. Gastroenterology 145, 293–308. 10.1053/j.gastro.2013.05.050 23751777 PMC3919211

[B137] Ventham N. T. Kennedy N. A. Adams A. T. Kalla R. Heath S. O'leary K. R. (2016). Integrative Epigenome-wide Analysis Demonstrates that DNA Methylation May Mediate Genetic Risk in Inflammatory Bowel Disease. Nat. Commun. 7, 13507–13514. 10.1038/ncomms13507 27886173 PMC5133631

[B138] Vileigas D. F. Harman V. M. Freire P. P. Marciano C. L. C. Sant'Ana P. G. de Souza S. L. B. (2019). Landscape of Heart Proteome Changes in a Diet-Induced Obesity Model. Sci. Rep. 9, 18050. 10.1038/s41598-019-54522-2 31792287 PMC6888820

[B139] W.H. Organization (2016). Global Report on Diabetes. Geneva, Switzerland.

[B140] W.H. Organization (2019). World Health Statistics 2019: Monitoring Health for the SDGs, Sustainable Development Goals. Geneva: World Health Organization.

[B141] Wang Y. Navin N. E. (2015). Advances and Applications of Single-Cell Sequencing Technologies. Mol. Cel. 58, 598–609. 10.1016/j.molcel.2015.05.005 PMC4441954 26000845

[B142] Wang D. Eraslan B. Wieland T. Hallström B. Hopf T. Zolg D. P. (2019). A Deep Proteome and Transcriptome Abundance Atlas of 29 Healthy Human Tissues. Mol. Syst. Biol. 15, e8503. 10.15252/msb.20188503 30777892 PMC6379049

[B143] Wang R. Li B. Lam S. M. Shui G. (2020). Integration of Lipidomics and Metabolomics for In-Depth Understanding of Cellular Mechanism and Disease Progression. J. Genet. Genomics 47, 69–83. 10.1016/j.jgg.2019.11.009 32178981

[B144] Wang C. A. Attia J. R. Lye S. J. Oddy W. H. Beilin L. Mori T. A. (2021). The Interactions between Genetics and Early Childhood Nutrition Influence Adult Cardiometabolic Risk Factors. Scientific Rep. 11, 1–13. 10.1038/s41598-021-94206-4 PMC8295375 34290306

[B145] Weber M. Hellmann I. Stadler M. B. Ramos L. Pääbo S. Rebhan M. (2007). Distribution, Silencing Potential and Evolutionary Impact of Promoter DNA Methylation in the Human Genome. Nat. Genet. 39, 457–466. 10.1038/ng1990 17334365

[B146] Weihrauch-Blüher S. Schwarz P. Klusmann J.-H. (2019). Childhood Obesity: Increased Risk for Cardiometabolic Disease and Cancer in Adulthood. Metabolism 92, 147–152. 30529454 10.1016/j.metabol.2018.12.001

[B147] Whitaker J. W. Boyle D. L. Bartok B. Ball S. T. Gay S. Wang W. (2015). Integrative Omics Analysis of Rheumatoid Arthritis Identifies Non-obvious Therapeutic Targets. PloS one 10, e0124254. 10.1371/journal.pone.0124254 25901943 PMC4406750

[B148] Wojcik G. L. Graff M. Nishimura K. K. Tao R. Haessler J. Gignoux C. R. (2019). Genetic Analyses of Diverse Populations Improves Discovery for Complex Traits. Nature 570, 514–518. 10.1038/s41586-019-1310-4 31217584 PMC6785182

[B149] Xue A. Wu Y. Zhu Z. Zhang F. Kemper K. E. Zheng Z. (2018). Genome-wide Association Analyses Identify 143 Risk Variants and Putative Regulatory Mechanisms for Type 2 Diabetes. Nat. Commun. 9, 2941. 10.1038/s41467-018-04951-w 30054458 PMC6063971

[B150] Ye Y. Chen X. Han J. Jiang W. Natarajan P. Zhao H. (2021). Interactions between Enhanced Polygenic Risk Scores and Lifestyle for Cardiovascular Disease, Diabetes, and Lipid Levels. Circ. Genomic Precision Med. 14, e003128. 10.1161/circgen.120.003128 PMC7887077 33433237

[B151] Zhang A.-h. Qiu S. Xu H.-y. Sun H. Wang X.-j. (2014). Metabolomics in Diabetes. Clinica Chim. Acta 429, 106–110. 10.1016/j.cca.2013.11.037 24321733

[B152] Zhou T. Zhang R. Ma J. (2021). The 3D Genome Structure of Single Cells. Annu. Rev. Biomed. Data Sci. 4. 10.1146/annurev-biodatasci-020121-084709 34465168

